# Incorporation of Natural Blueberry, Red Grapes and Parsley Extract By-Products into the Production of Chitosan Edible Films

**DOI:** 10.3390/polym13193388

**Published:** 2021-10-01

**Authors:** Simona Dordevic, Dani Dordevic, Petr Sedlacek, Michal Kalina, Karolina Tesikova, Bojan Antonic, Bohuslava Tremlova, Jakub Treml, Marcela Nejezchlebova, Lukas Vapenka, Ales Rajchl, Monika Bulakova

**Affiliations:** 1 Department of Plant Origin Food Sciences, Faculty of Veterinary Hygiene and Ecology, University of Veterinary Sciences Brno, Palackeho tr. 1946/1, 61242 Brno, Czech Republic; dordevicd@vfu.cz (D.D.); tesikovak@vfu.cz (K.T.); H20364@vfu.cz (B.A.); tremlovab@vfu.cz (B.T.); 2 Faculty of Chemistry, Brno University of Technology, Purkynova 118, 61200 Brno, Czech Republic; sedlacek-p@fch.vut.cz (P.S.); kalina-m@fch.vut.cz (M.K.); 3 Department of Molecular Pharmacy, Faculty of Pharmacy, Masaryk University, Palackeho tr. 1946/1, 61200 Brno, Czech Republic; tremlj@pharm.muni.cz (J.T.); nejezchlebovam@pharm.muni.cz (M.N.); 507342@mail.muni.cz (M.B.); 4 Department of Food Preservation, University of Chemistry and Technology Prague, Technicka 5, 16628 Prague, Czech Republic; lukas.vapenka@vscht.cz (L.V.); ales.rajchl@vscht.cz (A.R.)

**Keywords:** antioxidant activity, FTIR, barrier properties, antimicrobial properties

## Abstract

The aim of the research was to produce edible packaging based on chitosan with the addition of various concentrations of extracts of blueberry, red grape and parsley marcs. Packaging was made from extrudate extracts, which were subsequently analyzed by physicochemical methods: zeta-potential, gas barrier properties, thickness, water content, solubility, swelling degree, textural properties, total polyphenol content (TPC), polyphenols by high pressure liquid chromatography (HPLC), antioxidant activity, attenuated total reflectance Fourier-Transform spectroscopy (FTIR), antimicrobial activity and determination of migration of bioactive substances. The results indicate that a higher content of plant extracts have a statistically significant (*p* < 0.05) influence on properties of experimentally produced edible films. Edible films produced with the highest concentrations of red grapes marc extracts showed the most advantageous properties since antimicrobial activity against *E. coli* were the highest in this kind of produced film. The physical properties of edible films were also improved by the addition of extracts; gas permeability toward oxygen can be defined as advantageous, as can swelling degree, which decreased with higher concentrations of extracts. The research emphasized the possibility to use plant foodstuffs by-products in the production of edible/biodegradable films, helping in the overall sustainability and eco-friendliness of food/package production.

## 1. Introduction

The production of edible packaging based on polysaccharides has been developing greatly in recent years. With the use of edible—and therefore degradable—materials, it is possible to reduce the production of waste significantly [1], which is in line with the modern trends of a sustainable economy. There are many possibilities how to prepare the edible packaging and there are a lot of different compositions that can be used. The research concerning edible/biodegradable packaging can be focused on packaging production itself or it can be focused on edible/biodegradable packaging application on different food commodities. Recent publication was focused on the preparation of edible packaging from whey protein isolate nanofibers and carvacrol; the experimentally produced packing was applied on duck egg yolk and shelf life was monitored [2]. In another study, the packaging based on microalgal exopolysaccharides with the addition of red seaweed extract were applied on shrimps [3]. The technology of preparation of films significantly affected the properties of edible/biodegradable packaging; the different preheating temperatures were studied for the preparation of films based on soy protein isolate and soy oil that led to the changes of hydrophobicity [4].

Chitosan (N-deacetylated derivative of chitin) belongs to the film-forming components that are most often proposed for use in the production of edible packaging. As the second most abundant polysaccharide in nature, chitosan has become commonly used in various industries in the last decades, and many studies dealing with its further application potential, based, among others, also on its film-forming properties, have been published [5]. Its advantage is that it is a natural polysaccharide; thus, in contrast to synthetic polymers, it is biocompatible and biodegradable. Chitosan is also non-toxic, and it possesses antimicrobial properties [6,7]. Examples of chitosan use are its incorporation in wound healing products [8] and in water purification to remove mercury [9].

Byproducts from the food production are most often incorporated in some low value products, for example, in feed production. However, as these products are still rich in various bioactive substances, it seems more reasonable to utilize them as active ingredients, providing the material with added value, such as in the production of edible packaging. This way, the packaging material can be provided with highly valued properties such as the shelf life improvement of packaged food, oxidation and dehydration prevention, etc. These additional properties occur mainly due to the ability of the packaging material to release the bioactive components to the packaged food in a gradual and controlled manner. Apart from the active performance, edible packaging can also have intelligent properties, where the packaging can act as an indicator of a change in the condition of the packaged food (an example might be a change in the color of the packaging during food spoilage) [10,11,12]. Numerous works have already dealt with a use of the food industry byproducts in the production of edible packaging. For instance, the use of protein and pectin extracted from pumpkin by-products (seeds and skins) has been investigated [13], as well as the incorporation of hydrolyzed gelatin obtained from carp skin, which may show some antioxidant activity [14,15], same as the use of mango seed core extract [16].

There are also other food industry byproducts that can be utilized in the production of food packaging. Blueberries, for instance, represent a very good source of anthocyanins that can be used in the food industry as a substitute for synthetic dyes, because dyes found in nature are safer compared to synthetic dyes. Among the most interesting properties of anthocyanins are their color change in environments with different pH and their antioxidant properties [17]. These substances could also replace the currently commonly used synthetic antioxidants BHA (butylhydroxyanisole) and BHT (butylhydroxytoluene) since they have adverse effects on enzymes found in the human body [18]. Red grapes are known for their high content of polyphenols. The group of flavan-3-ols found in red grapes includes catechin, epicatechin, gallocatechin and epigallocatechin [19]. In general most polyphenols in grapes are found in the skin and seeds [20]. Parsley has antibacterial, antiviral, anti-inflammatory and antioxidant properties [21], and it contains flavonoids and coumarins [22].

In the previous research the sensory analysis, color characterization, microscopy and biodegradability were studied [23]. The aim of the research was to experimentally produce edible packaging with the incorporation of plant byproducts (blueberries, red grapes and parsley marc) and to evaluate their physical and chemical properties; experimentally produced films are comprehensively analyzed, and based on these results potential applications can be found.

## 2. Materials and Methods

### 2.1. Materials

Low molecular weight chitosan (50,000–190,000 Da), as well as other chemicals needed to perform the analyses, were purchased from Sigma-Aldrich (St. Louis, MO, USA) Parsley (*Petroselinum crispum* (Mill.) A. W. Hill) was grown in the Czech Republic, blueberries (*Vaccinium myrtillus* L.) were grown in Spain and seedless red grapes (*Vitis Vinifera* L. Crimson Seedless variety) were grown in Chile. All the above-mentioned plant raw materials were purchased in the Tesco store in Brno, in the Czech Republic.

### 2.2. The Extract Preparation

Extracts were prepared from the byproducts after preparation of juices; the residual waste after juicing was collected. The first step was the juicing of raw materials, the resulting waste containing part of the pulp and husks was obtained. A total of 10 g of the by-product thus obtained were weighed into a beaker and poured into 100 mL of hot distilled water (100 °C) and infused for 10 min and then the extract was filtered and used to produce edible packaging.

### 2.3. Edible Packaging Preparation

The preparation of edible films (Table 1) included following: 1.5 g of low molecular weight chitosan was weighed into a 250 mL beaker and subsequently dissolved in 1% lactic acid. The amount of 1% lactic acid solution varied depending on the addition of the extract (the amount of lactic acid was replaced by the extract). An amount of 135 mL of 1% lactic acid was used in the samples without extract addition. The samples were then transferred to magnetic stirrers where they were stirred for 15 min at 50 °C and at 500 rpm. The fresh prepared plant extract was then added at concentrations of 5%, 10% and 20% (*w*/*w*), followed by stirring for five minutes and the addition of glycerol as a plasticizer. After five minutes, the film-forming solution was poured into 150 mm diameter Petri dishes and left to dry for 48 h.

### 2.4. Stability of the Film-Forming Solutions

#### Zeta Potential

The zeta potential of chitosan in all above mentioned film-forming solutions was determined by Zetasizer Nano ZS (Malvern Panalytical, UK) by means of the electrophoretic light scattering method. The values of zeta potential were determined through the measurement of the electrophoretic mobility of particles in used dispersion media after the application of an external electric field. For purposes of the analyses, approximately 1 mL of individual liquid film-forming solutions were transferred into the spectroscopic cuvette (optical glass, 12 × 12 × 45 mm, PSC 1115, Malvern Panalytical, UK), and subsequently the universal dip cell (Zen 1002, Malvern Panalytical, UK) was immersed to be able to apply the external electric field (used effective voltage 4.890 ± 0.011 V). The analysis of one sample was performed in 5 repeated measurements (each measurement represents an average value of 12 scans).

### 2.5. Basic Morphological and Textural Properties of the Films

#### 2.5.1. Film Thickness

Film thickness was measured using a Mitutoyo M310-25 micrometer (Kawasaki, Japan) at 5 different locations.

#### 2.5.2. Textural Properties

Strength (MPa) and breaking strain (%) were measured using a TA.XT plus texturometer (Godalming, UK) by the ASTM International Test Method—ASTM D882-02. The produced packages were cut into rectangles measuring 1 × 5 cm and each measurement was performed 5 times.

#### 2.5.3. Gas Barrier Properties

Water vapor transmission rate (WVTR) was determined by gravimetrical method according to DIN 53 122 standard at 23 °C and relative humidity of 85%. Five parallel samples were tested for each packaging material.

Oxygen permeability was determined using OxTran 2/20 MH measuring system (MOCON Inc., Minneapolis, MN, USA) according to ASTM D3985—17 standard at 23 °C and relative humidity of 0%. Two parallel samples were tested for each packaging film.

### 2.6. Basic Compositional and Structural Analysis

#### 2.6.1. Water Content, Solubility and Swelling Degree

The determination was performed according to the slightly modified method published by Souza et al. [24]. The film samples were cut into 2 × 2 cm squares and then weighed on an analytical balance (KERN, Germany), the weight was marked as W1. Subsequently, the films were placed in an oven (Ecoccel 55) for 2 h at 105 °C and then reweighed (W2). Subsequently, the samples were placed in beakers containing 25 mL of water and, after 24 h at room temperature, dried and reweighed (W3). Next, they were transferred to an oven for 24 h at 105 °C and then weighed (W4). Replicates (*n* = 6) were prepared for each sample. The results were obtained from the following equations:

Water content (%) = [(W − W2)/W1)] × 100 Solubility (%) = [(W2 − W4)/W2] × 100 Swelling degree (%) = [(W3 − W2)/W2] × 100(1)

#### 2.6.2. Attenuated Total Reflectance Fourier-Transform spectroscopy

Fourier transform infrared (FTIR) spectra of the prepared films were with an iS50 FTIR spectrometer (Thermo Scientific, Waltham, MA, USA). All measurements were taken from a surface of a film at ambient temperature (in an air-conditioned room) with the built-in single-reflection diamond attenuated total reflectance (ATR) crystal. An individual absorption spectrum was collected as an average of 16 scans with a resolution of 4 cm^−1^ (data spacing 0.5 cm^−1^). Each film was analyzed at 6 randomly distributed spots on its surface (3 spots on each—back and front—side of the film), FTIR spectra are provided in respective figures, representing an average of spectra collected for an individual sample. The whole-spectra PCA analysis was performed using a standard multivariate principle component program written in-house using MATLAB software (MathWorks, Natick, MA, USA) at Institute of Scientific Instruments, Czech Academy of Sciences [25].

### 2.7. Content of the Antioxidant Compounds in the Films

#### 2.7.1. Attenuated Total Reflectance Fourier-Transform Spectroscopy

The total polyphenol content was measured using the Folin–Ciocalteu method described by Tomadoni et al. [26] with slight modifications. An amount of 1 g of the edible packaging was weighed into a beaker and then 40 mL of distilled water was added. The samples were stirred for 10 min and then 1 mL was taken into a 25 mL volumetric flask, 5 mL of Folin–Ciocalteu solution (diluted 1:10 by volume) and 4 mL of 7.5% Na_2_CO_3_ were added to the sample. The samples were incubated in the dark for 30 min. The absorbance was measured at 765 nm against a blank (1 mL of the sample was replaced by 1 ml of distilled water). The results were expressed as the content of gallic acid per gram of the sample. Each sample was measured in triplicate. 

#### 2.7.2. HPLC—Polyphenolic Compounds Determination

HPLC chromatograph, 1260 Infinity high performance liquid chromatograph (Agilent Technologies, Santa Clara, CA, USA) was used to determine polyphenolic compounds in the experimentally produced edible packaging. The method of Gómez-Estac et al. [27] with slight modifications was used. The mobile phase consisted of 1% phosphoric acid (A) and acetonitrile (B) in the following composition: 80% of A and 20% of B for 20th min, 70% of A and 30% of B from 20th to 25th min, 60% of A and 40% of B from 25th to 40th min. The separation was performed on a Zorbax SB-C18 4.6 × 250 mm column (Agilent Technologies, Santa Clara, CA, USA) (the temperature was 25 °C) and detection was performed on a DAD array detector (detection wavelength was 324.5 nm) The injection volume was 10 µL. Each sample was measured in triplicate. The software used for HPLC chromatohraph was Agilent ChemStation.

### 2.8. Evaluation of the Antioxidant Properties of the Films

#### 2.8.1. FRAP (Ferric Reducing Antioxidant Power)

The FRAP was conducted by Behbahani et al. [28]. An amount of 0.1 g of the sample was weighed, to which 20 mL of 75% methanol was added, and the samples were then sonicated in a water bath for 30 min. Subsequently, 180 μL of the extract was pipetted into dark vials, to which 300 μL of distilled water and 3.6 mL of working solution (acetate buffer, TPTZ (2,4,6-Tripyridyl-S-triazine) and FeCl_3_) were added. The samples were further incubated for 8 min in the dark. Absorbance was measured at 593 nm against a blank sample (distilled water + working solution). Trolox was used to prepare a calibration curve and the results were expressed as μmol of Trolox per gram of sample. Each sample was measured in triplicate.

#### 2.8.2. ABTS (2,2′-Azino-Bis(3-Ethylbenzothiazoline-6-Sulfonic Acid))

The ABTS method was conducted according to Thaipong et al. [29] with slight modification. An amount of 0.1 g of the sample was weighed into dark vials, to which 20 mL of ethanol was added, and the samples were sonicated for 30 min. Then, 12 to 16 h before the measurement, 10 mL of 0.007M ABTS solution was mixed with 10 mL of 0.00245 M potassium persulphate solution. The solution was diluted before the measurement so that its final absorbance at 735 nm was 0.7. Then, 1980 μL of ABTS solution was mixed with 20 μL of the prepared package extract. The samples were incubated for 5 min in the dark and then the absorbance at 735 nm was measured. Each sample was measured in triplicate. The results were calculated according to the following formula:

ABTS [%] = [(Abs_ABTS_-Abs_sample_)/Abs_ABTS_] × 100 (2)

Abs_ABTS_—absorbance of ABTS solution (−). Abs _sample_—absorbance of sample (−).

#### 2.8.3. DPPH (2,2-Diphenyl-1-Picrylhydrazyl)

The DPPH method was conducted according to Adilah et al. [30], with slight modifications. The 0.1 g of the film sample was weighted and 20 mL of ethanol was added, the samples were sonicated for 30 min, then the extracts were filtrated, 3 mL of extract and 1 mL of 0.1 mM DPPH solution in ethanol were mixed. The samples were incubated at laboratory temperature in the dark for 30 min and the absorbance was measured at 517 nm by spectrophotometer (CE7210 DIET-QUEST, Cambridge, England). Each sample was measured in triplicate. The scavenging activity of DPPH was calculated according to the following formula:

DPPH_scavenging activity_ [%] = [(Abs_DPPH_ − Abs_sample_)/Abs_DPPH_] × 100 (3)

Abs_DPPH_—absorbance of DPPH solution (−), Abs _sample_—absorbance of sample (−).

### 2.9. Antimicrobial Properties of Films

Edible films were exposed to UV radiation (wavelength 260 nm) due to physical disinfection. Subsequently, disks (edible films) with a diameter of 5 mm were cut in an aseptic environment. A modified disk diffusion method according to EUCAST (European Committee on Antimicrobial Susceptibility Testing) was used to determine the antimicrobial resistance of edible films. *Staphylococcus aureus* subsp. *aureus* (methicillin resistant strain) CCM 7110 and *Escherichia coli* CCM 3954 were cultivated with edible coatings, solid medium according to Mueller and Hinton (MUELLER-HINTON broth, Agar for microbiology, Sigma-Aldrich) was used and solid medium was used for culturing *Candida albicans* CCM 8261 with edible coatings, malt broth (Malt Extract Broth, Agar for microbiology, Sigma-Aldrich). The inoculum concentration was adjusted to approximately 1−2 × 10^8^ CFU/mL, corresponding to 0.5 degree McFarland turbidity standard. An amount of 1 mL of inoculum was spread on the surface of the agar and, after drying, 4 discs from edible films were placed on a 9 cm diameter dish. Each set of films (LBO, LPE and LHR) was tested on all three microorganisms and each plate included a CH_L_ control in addition to the individual discs set. The inoculated plates were incubated for 18 h at 35–37 °C. A positive result was considered in the case when the microorganism did not outgrow the disc of the edible shell or even created an inhibition zone around the disc in which the tested microorganism did not grow. Depending on the used medium, in some cases the edible casing flowed during the cultivation at 35–37 °C.

Reference strains of microorganisms *Staphylococcus aureus* subsp. *aureus* (MRSA) CCM 7110, *Escherichia coli* CCM 3954 and *Candida albicans* CCM 8261 were obtained from the Czech Collection of Microorganisms, the Department of Experimental Biology, Faculty of Science, Masaryk University.

### 2.10. Determination of Migration of Bioactive Compounds

The films were cut into 1 × 1 cm squares and immersed in 2.5 mL of a 10% aqueous ethanol solution, which was chosen as simulant A according to the Regulation No. 10/2011 on plastic materials and other materials intended to come into contact with food [31]. The samples were then incubated for 10 days at 40 °C, conditions for using the food packaging from 3 to 30 days at 20–40 °C. The samples were then analyzed on the following analysis: FRAP, DPPH, ABTS and the total content of polyphenols, which were chosen as indicators for the migration of substances that may affect the shelf life of packaged foods.

### 2.11. Statistical Analysis

All results in tables present mean values ± standard deviations. Statistical significance of *p* < 0.05 was determined by a one-sample ANOVA test using parametric Tukey’s test (when Leven’s test showed *p* > 0.05) and non-parametric Games–Howell post hoc test (when Leven’s test showed *p* < 0.05). IBM SPSS software was used for statistical processing.

## 3. Results and Discussion

### 3.1. Stability of the Film-Forming Solutions

The behavior of chitosan in studied film-forming solutions was initially analyzed through the determination of average zeta potential in the individual used samples. The results are shown in Table 2. Chitosan as a representative of polycationic polysaccharides [32] formed positively charged particles in all used dispersion media. The explanation is straightforward, the positive charge of observed particles is caused by the protonation of chitosan amino groups in used acidic solutions (caused mainly by the presence of 1% lactic acid) as the isoelectric point of chitosan is present at pH around 6; the pH of film forming solutions was 3.20 ± 0.04 [6]. The concentration of chitosan in the samples was constant. It was found that even with a change in the dispersion medium (ratio of lactic acid solution and plant extract) the values of zeta potential of the particles were not affected. The determined values of zeta potential showed statistically significant differences in some samples (*p* < 0.05), but it must be emphasized that the results are very similar numerically. This conclusion is supported also by similar measured conductivities of all the samples, indicating the comparable content of dissolved low-molecular ions in the individual used dispersion media but, primarily, also the similar colloidal behavior of chitosan in these media. 

Another important fact which can be observed from the zeta potential measurement is the stability of the prepared colloidal dispersion. All the determined values of zeta potential are above 30 mV, which indicate high stability of chitosan particles against mutual aggregation of particles for all the used dispersion media. Besides the mentioned high stability of described sample, these prepared systems can also be considered as well-dispersed systems from the colloidal point of view [33].

### 3.2. Basic Morphological and Textural Properties of the Films

The thickness of films is given in Table 3. No net decrease or increase in thickness was found in the samples with increasing addition of the extract, these values fluctuated in the samples. The results also do not show statistically significant differences (*p* > 0.05), so it can be said that the increasing percentage of extract did not affect the thickness of the resulting package. In the case of comparison with previous studies, there was both a significant increase in thickness values after the addition of plant extracts [15,34] but there were also findings that resulted in a decrease [35,36].

The results of textural properties are shown in Table 4. The interaction of hydrocolloids and other additives such as plasticizers, water and antimicrobial substances has the greatest influence on the textural properties of edible coatings [37]. The 5CH_LBO_, 5CH_LPE_, 5CH_LHR_ and 10CH_LHR_ samples are statistically significantly different (*p* < 0.05) from the 20CH_LPE_ sample that had the highest strength (0.10 ± 0.02 MPa). No statistically significant difference (*p* > 0.05) was found between the other samples. The comparison of results with the control sample CH_L_ did not resulted in statistically significant differences (*p* > 0.05) between the samples with the addition of extracts. The increase in strength can be caused by interactions between plant extracts that contain phenolic acids and their esters. These compounds can react with the hydrophilic groups present in the chitosan matrix, and this interaction can lead to stronger adhesion between the plant extracts and the chitosan molecules. These interactions can cause an increase in strength [38]. In previously published articles, it has been found that some additives incorporated into the chitosan matrix both increase and decrease the strength of the prepared packages. The explanation is similar to that described above: the increase in strength is due to stronger interactions between additives and chitosan, and the decrease in strength is due to weak interactions [39].

The prepared chitosan packaging was characterized by high flexibility, which was observed by the handling of the packaging itself and subsequently confirmed by measuring the flexibility, where the results are presented in Table 2. Flexibility decreased with the addition of the extract. Compared to the CH_L_ sample, the lowest value of elasticity was found in the 20CH_LBO_ sample, but no statistically significant difference was found (*p* > 0.05). The results correspond to the results of the force; due to the interaction between phenolic acids and chitosan, the flexibility is not so high in most samples [38]. It has also been found, when compared with the results of measuring the strength and elasticity of packages made of κ-carrageenan and ι-carrageenan with the addition of lapacho tea extract, that chitosan packages are more flexible and also have a lower strength value [40].

The results of gas barrier properties are summarized in Table 5. Water vapor transmission rate of all tested samples was higher than 1000 g/m^2^ day, which is a relatively high value that limits the use of this material for packaging products that need to be protected from moisture. However, this value is similar for chitosan-based materials [41,42,43]. Similar values of WVTR were measured for bleached Kraft paper [44]. The results also showed that the addition of plant extracts increased the WVTR value of the chitosan-based material by an average of: 3.84 ± 1.14% for 5% addition of blueberry extract, 9.63 ± 0.23% for 5% addition of parsley extract, 8.05 ± 1.64% for 5% addition of red grapes extract. The lowest average value of WVTR for the material with the addition of plant extract 1270.0 ± 25.3 g/m^2^ day was achieved for film with 5% addition of blueberry extract, the highest average value of 1724.6 ± 30.8 g/m^2^ day was achieved for material with 20% addition of red grapes extract.

Changes of oxygen permeability caused by the addition of plant extract are opposite to the changes of WVTR according to the plant extract addition. The reduction in oxygen permeability compared to chitosan films without the addition of plant extract was on average 21.30 ± 3.15% for 5% addition of blueberry extract, 16.11 ± 1.70% for 5% addition of parsley extract and 13.85 ± 3.97% for 5% addition of red grapes extract. The lowest average value of oxygen permeability of the material with the addition of plant extract 4.1 mL/m^2^ day 0.1 MPa was measured for the material with 20% addition of blueberry extract, the highest average value of 13.7 mL/m^2^ day 0.1 MPa was achieved for a film with 5% addition of red grapes extract. The resulting values are relatively low and correspond to permeability values for commonly used packaging materials based on, e.g., polyamide, polyethylene terephthalate [45].

Similar and completely different effects of the addition of plant extracts on the barrier properties of final materials have been published [46,47]. The increase in permeability may be caused by the destabilization of the original chitosan matrix by extract components that may act as plasticizers [48]. The reduction in permeability is then explained mainly by the possibility of crosslinking between components of the extracts and the polymer matrix [49].

For the purposes of wider use of this material in food packaging, it would be good to combine the material with barrier materials preventing the penetration of moisture into the packaged product. An example may be the incorporation of waxes, oils, etc., directly into the material [47,50]. A disadvantage of this method may be a significant reduction in oxygen transmission rate [48].

### 3.3. Basic Compositional and Structural Analysis

Data for water content, solubility and swelling degree are summarized in Table 6. In the case of water content, a decrease in water content was observed in the samples after the addition of the extracts, but significant (*p* < 0.05) differences in comparison with the control sample (CH_L_) were noticed among the following samples: 10CH_LBO_, 5CH_LPE_ and 20CH_LPE_. The reduction of the water content in the packaging with the addition of extracts is due to the formation of hydrogen bonds, which in turn reduce the availability of hydroxyl groups and amino groups and thus limit the interaction of chitosan with water [51,52].

The solubility results did not differ significantly with the obtained values, and it can therefore be solved that the addition of extracts from red grape, blueberry and parsley pomace does not affect the solubility. Similar results were found in research by Bourbon et al. [53].

In the analysis of swelling degree, the highest values were reached in the case of the CH_L_ sample (the control sample without the addition of extract), in the samples where extracts were added, the values of the swelling degree decreased, but in comparison with CH_L_ the differences were not statistically significant (*p* > 0.05). In all other samples there was a gradual decrease in the value of the swelling degree, but only between the samples 5CH_LHR_ and 20CH_LHR_ was a statistically significant (*p* < 0.05) difference found. For the swelling degree, the reduction is caused similarly to the water content. When the presence of polyphenolic substances blocks the active groups of chitosan available for water adsorption [24], these results are confirmed because for samples with the lowest swelling degree (20CH_LBO_ and 20CH_LHR_), the highest value of the content of polyphenolic substances were found (see section *Content of the Antioxidant Compounds in the Films*).

**Figure 1:**
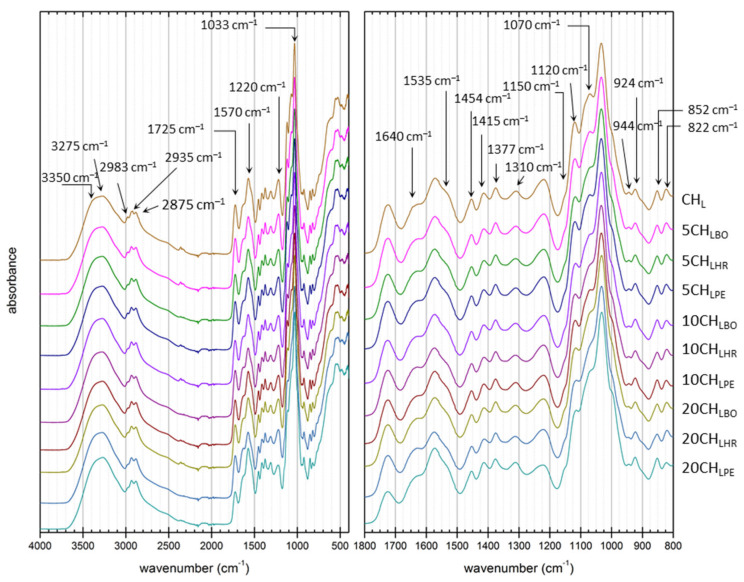
ATR-FTIR spectra of all prepared films. Every spectrum represents an average of spectra recorded separately on six randomly distributed spots of the film surfaces (three on each side).

Furthermore, the Fourier transform infrared spectroscopy was used to provide a closer look on how the addition of plant extracts alters the chemical structure of the prepared chitosan film. The FTIR spectra of all analyzed films are shown in Figure 1. It can be seen that the presence of a plant extract does not induce any distinct spectral features (such as the occurrence of new absorption bands) that could be directly assigned to the molecular structure of the extract components. In other words, the structure of the films as observed by FTIR is primarily governed by the matrix composition of the film. In this structure, characteristic FTIR vibrations that correspond to the three matrix components can be found in all spectra. Firstly, chitosan as the main film-forming components is reflected by: (i) absorptions originating from amide linkage in acetylated amine groups, in particular C=O stretch in amides at 1640 cm^−1^ (referred to as Amide I), N-H in-plane bend at 1535 cm^−1^ (Amide II), C-N stretch at 1310 cm^−1^ (Amide III), (ii) vibrations attributed to deacetylated amine groups (NH_2_ bend at 1570 cm^−1^, less pronounced N-H stretches at 3350 and 3270 cm^−1^ overlapped by intensive -OH stretch of all matrix components and moisture), and (iii) vibrations of the oxygen containing groups, namely asymmetric C-O-C stretching in glycosidic bond (1150 cm^−1^) and C-O stretching at 1070 and 1030 cm^−1^. Secondly, presence of lactic acid is manifested by: (i) characteristic bands of carboxylic groups (C = O stretch in carboxylic groups at 1725 cm^−1^, less intensive shoulder of H-bonded dimers at 2600 cm^−1^, C-O stretch at 1220 cm^−1^) and (ii) methyl vibrations (clearly visible asymmetric C-H stretch in CH_3_ at 2983 cm^−1^, symmetric “umbrella” bend at 1377 cm^−1^). Last but not least, glycerol presence is reflected by the (i) characteristic absorption of -CH_2_- groups (asymmetric and symmetric C-H stretches at 2935 and 2875 cm^−1^, respectively, CH_2_ scissoring bends at 1454 and 1415 cm^−1^) and (ii) contribution to −OH related vibrations in 3000–3500 cm^−1^ (O-H stretches) and in 1150–800 cm^−1^ (C-O stretches), where the presence of glycerol is usually accompanied by characteristic asymmetric stretches at 924 and 852 cm^−1^.

**Figure 2:**
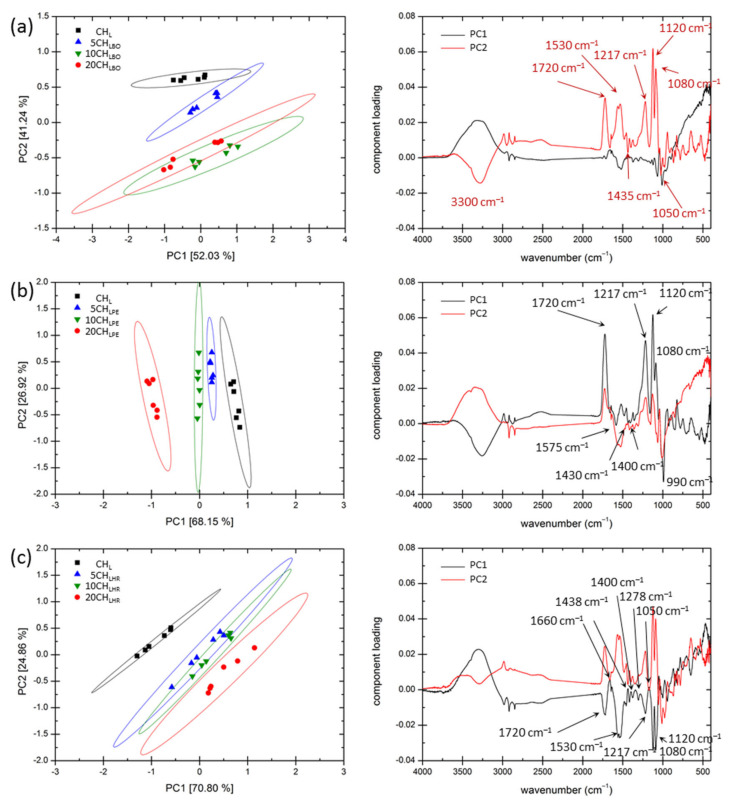
Results of Principal Component Analyses of the sets of packaging films prepared without and with various content of: (**a**) blueberry extract, (**b**) parsley extract, and (**c**) red grapes extract. The PCA was performed with all measured spectra (6 for each of the films). The value in the square bracket represents the relative variance that is composed in the respective principal component. Ellipses represent the 95% interval of confidence.

At first sight, it may seem that the addition of plant extracts has negligible effect and may not be observed in the FTIR spectra. However, the effect of the extracts on the FTIR spectra may be revealed when an advanced processing is applied on the spectra. We have used the whole-spectra Principal Component Analyses for this purpose. In this technique the set of original variables (absorbances measured at individual wavenumbers) is replaced by the new set of variables (principal components, calculated as specific linear combinations of the original variables) with the same total variance (i.e., the same overall information on the observed system) but with different spread of the information among these variables. In order to reveal the effect of individual extracts, three separate Principal Component Analyses were performed for the three sets of films with a particular extract added. The results of these analyses are shown in Figure 2 in form of the respective two-dimensional factor planes of the two principal components that covers the highest relative variance (PC1 and PC2) together with their spectral loadings. The loading shows how the component is composed from the original data, in other words, it illustrates what are the main spectral variations among the analyzed spectra from the view of this component.

Apparently, PCA analysis revealed the spectral features that clearly distinguish FTIR spectra of different films. In Figure 2a, it can be seen that the clusters that represent films with different content of the blueberry extract are separated in the plot mainly via component PC2, whereby the increasing content of the extract results in a more negative value of this component. From the loading of this component, it can be seen that an increase of the content of blueberry extract is reflected in the spectrum mainly by a decrease in the signal of lactic acid (note the positive loading of PCA at 1720, 1217, 1120 and 1180 cm^−1^) or its salt (positive loading of an asymmetric stretch of -COO- at 1530 cm^−1^). On the other hand, in the negative loading of PC2 that correlate with the content of the blueberry extract, spectral features can be found that were previously ascribed to anthocyanin, such as absorption at 1435 cm^−1^ (C-N in anthocyanin) or the flavonoid C-O-C stretch at 1070 cm^−1^ [54]. Similarly, also the clusters that represent PCA coordinates of the films prepared with different contents of parsley extract are well separated in the PC1-PC2 coordinate plot (see Figure 2b), mainly distinguished by the value of PC1 component. Once again, from the loading of this component, a negative correlation between the contents of the extract and of the lactic acid is evident. The most prominent spectral features in negative loading of this component (marked in Figure 2b) well correspond with the vibrations that were already found in parsley essential oil [55]. Finally, Figure 2c shows results of PCA for the films prepared with different content of red grapes extract. Clusters representing these films are once again well separated from the CH_L_ matrix film. Again, increase in the extract is linked with decrease of the signal of lactic acid and lactate in FTIR spectra of the films (see the negative loading of PC1), while its increase enhances the signal (represented by the positive loading of PC1), which is in a good agreement with a spectrum published for the biostimulant prepared from red grapes recently [56].

### 3.4. Content of the Antioxidant Compounds in the Films

Phenols and phenolic acids are metabolites of plants with the highest antioxidant activity [57]. The content of phenolic acids in the experimentally produced packaging is summarized in Table 7. The CH_L_ sample was found to contain total polyphenols 0.12 ± 0.01 mg of gallic acid/g. Small amounts of polyphenols have also been found in previous research [58,59,60]. The measurement of content of a certain polyphenol in the package without the addition of extracts may have been due to the formation of chromogens that are formed by the reaction of Folin–Ciocalteu reagent with non-phenolic reducing agents and that can subsequently be detected by spectrophotometer measurements [59]. The addition of blueberry, parsley and grape by-products extracts showed an increase in content of phenolic acids in samples with extracts. Total polyphenols contents in the extracts used to make the packaging was as follows: blueberry extract 0.26 ± 0.00 mg gallic acid/mL; parsley extract 0.04 ± 0.00 mg gallic acid/mL; grape extract 0.12 ± 0.00 mg gallic acid/mL.

5CH_LBO_, 10CH_LBO_, and 20CH_LBO_ were statistically significantly different (*p* < 0.05) from CH_L_, meaning that the addition of blueberry extract has a high effect on the total polyphenol content. The addition of parsley by-product extract increased TPC, but a statistically significant difference (*p* < 0.05) was found only between the 20CH_LPE_ and CH_L_ samples, where the content of polyphenols in the 20CH_LPE_ sample was about half that of the 20CH_LBO_ and 20CH_LHR_ samples. Red grape by-product extract affected the total content of polyphenols the most, as the 20CHLHR sample had the highest content of polyphenols (0.96 ± 0.05 mg gallic acid/g), but no statistically significant difference (*p* > 0.05) was found between the 20CH_LHR_ and 20CH_LBO_ samples.

In previous research, a higher TPC content was found in packages with the addition of blueberry ash fruit extract, macadamia peel extract and banana peel extract [61], which may be affected by the procedure extract preparation—ratio of solid material and solvent, type of solvent and amount of addition to the matrix of film-forming solution [62].

Aside from the determination of total polyphenol content, the selected common representatives of the polyphenolic substances were directly assayed in the prepared films. The results of the determination of individual polyphenolic substances in the samples are given in Table 8 and Table 9. All samples were analyzed for the presence of rosemary acid, chlorogenic acid, as well as epigallocatechin, epicatechin gallate and epicatechin.

In the case of packages with the addition of plant extracts, it was found that rosemary acid is found in the highest concentration in samples with the addition of parsley extract, the sample 20CH_LPE_ reached a concentration of 0.0206 ± 0.0009 mg rosemarinic acid/g.

Chlorogenic acid was found at the highest concentrations in samples with the addition of blueberry extract (20CHLBO: 0.0781 ± 0.0061 mg/g), the result was statistically significantly different (*p* < 0.05) from the values in all other samples. Thus, it was confirmed that the extracts of moldings blueberries occurred from crossing of chlorogenic acid, compared with cranberries, black currants, strawberries, red currants, raspberries and blackberries, had the second highest value [63]. Thus, it was confirmed that the extracts of blueberries crossed with chlorgenic acid had the second highest content, as compared with cranberries, black currants, strawberries, red currants, raspberries, and blackberries [63].

Epigallocatechin was also found in most of samples with the addition of blueberry extract and similar results were obtained in the determination of epicatechin. Epicatechin gallate was found in the highest concentration in samples with the addition of red grape extract. Epigallocatechin and epicatechin belong to the group of flavan-3-ols that occur in red grapes [19].

### 3.5. Evaluation of the Antioxidant Properties of the Films

Aside from the determination and identification of the components with antioxidant activity, direct monitoring of antioxidant properties of the films was performed as well. These properties have been characterized by performing three different assays, each with different reaction conditions that can affect obtained results. Combination of three methods certainly provides more accurate perception about the experimentally produced edible packaging antioxidant properties. The obtained results confirmed that the addition of extracts increased the antioxidant properties of the chitosan films. The antioxidant activity of the extracts used to prepare the packaging samples was as follows: blueberry extract 0.176 ± 0.001 μmol Trolox/mL; 73.18 ± 0.44% ABTS; grape extract 0.021 ± 0.001 μmol Trolox/mL; 23.35 ± 0.19% ABTS; parsley extract 0.0 ± 0.0 μmol Trolox/mL; 6.32 ± 0.22% ABTS.

The results of the antioxidant properties are summarized in Table 10. The FRAP method showed the highest results for the 20CH_LBO_ sample (2.45 ± 0.06 μmol Trolox/g), where it must be emphasized that the addition of by-product extracts had a statistically significant impact (*p* < 0.05) on the FRAP method results, regardless of the addition concentration. The best antioxidant properties measured by the ABTS method were found in the sample 20CH_LHR_ (the sample with the highest antioxidant property, though, antioxidant properties of other samples can be defined as poor, accordingly) and similarly to the FRAP method, a statistically significant difference (*p* < 0.05) was found between all samples compared to CH_L_, except for the sample 5CH_LBO_. DPPH showed the same trend as FRAP and ABTS. The highest result was determined in the sample 20CH_LBO_ (5.39 ± 0.06%) and this result is statistically significantly different (*p* < 0.05) from all other DPPH results.

As expected, to some extent the antioxidant activity was also found in a control sample without the addition of extracts, where the main component is chitosan. Chitosan belongs to the compounds with the properties of inhibiting reactive oxygen species (ROS) and can prevent lipid oxidation in food, but is also in biological systems [64,65]. The antioxidant activity of edible packaging with the addition of extracts was higher because plant extracts are a good source of antioxidant compounds. In general, red fruits are very good sources of antioxidant compounds, as they contain anthocyanins that have these properties^16^. Higher antioxidant activity was also observed in packaging with the addition of red grape by-product extract. It is mentioned in the literature that if the extract from the marc is used in higher concentrations, then its antioxidant activity is comparable to synthetic BHT [66]. Parsley is also one of the plants with antioxidant properties [67], but in comparison with other samples, the antioxidant activity was sometimes up to half lower in packages with the addition of parsley extract.

### 3.6. Antimicrobial Properties of Films

**Figure 3:**
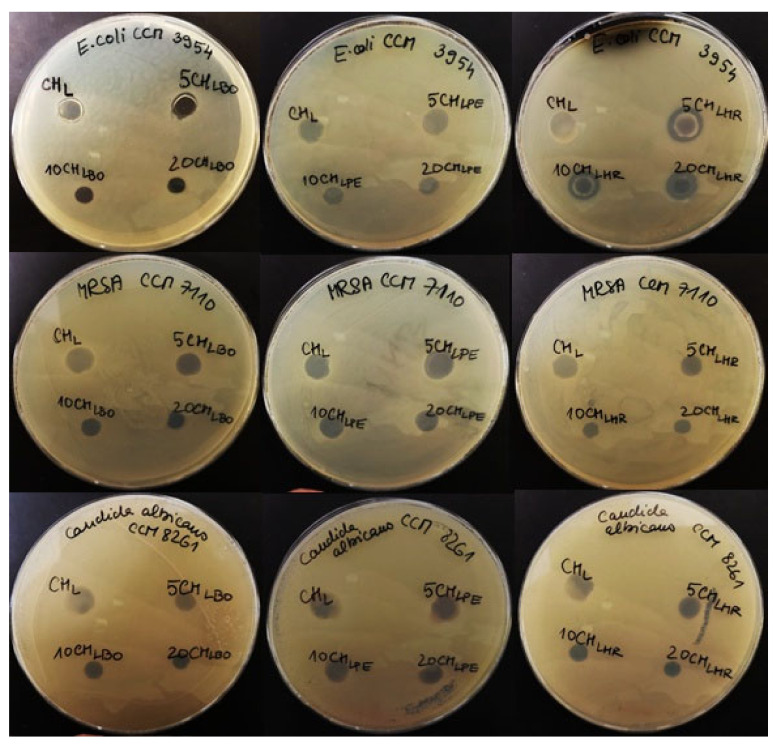
The results of antimicrobial activity of chitosan edible films.

The results of antimicrobial activity are presented in Figure 3. There was no overgrowth observed in all samples. These findings can be considered as a confirmation of antimicrobial effect of analyzed films. The highest antimicrobial efficiency showed the films with the addition of red grapes by-products extracts against Gram-negative microorganism *Escherichia coli* CCM 3954. The inhibition zones were observed in samples 5CH_LHR_ and 10CH_LHR_ (2 mm) and in the sample 20CH_LHR_ (2.5 mm). In the previous studies the antimicrobial activity of red grapes skin extract was evaluated and an antimicrobial efficiency was reported against all tested microorganisms [68,69]. The blueberry extracts were also analyzed in previous research. The reported antimicrobial activity was unfortunately not confirmed by our results. It should be stressed, however, that the same happened with the analysis of the total polyphenol content and antioxidant activity results. We assume that the results were probably not as high due to the lower level of active compounds in samples with the addition of by-products extracts compared with the samples with the addition of whole fruits/vegetable extracts. The antimicrobial properties of plants are caused mainly by the presence of phenolic compounds [70].

### 3.7. Release of the Active Components from the Films

According to the legislation, simulant A was chosen to determine the migration values of active substances, it is a simulation of the transition of active substances into foods with a hydrophilic character which, according to EU Regulation No. 10/2011, includes the following food commodities with the hydrophilic character: nuts in paste or cream, fresh vegetables, fish, fresh meat and processed meat products, fried potatoes, donuts, preparations for making soups, etc. The results of migration tests for manufactured packaging are shown in Table 11 (polyphenol content) and Table 12 (antioxidant properties). The values of the results of polyphenols showed that there is a migration of polyphenolic substances, which was confirmed by the analysis of antioxidant activity, where higher values were measured with the addition of extracts from by-products of blueberries, parsley and red grapes.

For polyphenols, the highest migration value was recorded for the 20CH_LHR_ sample (0.016 ± 0.000 mg gallic acid/mL), which is statistically significantly (*p* < 0.05) different from all other measurements of polyphenol migration.

In determining the antioxidant activity, the samples 20CH_LHR_, 20CH_LBO_ and 20CH_LPE_ achieved the best results, as described above, due to the higher concentration of extracts used for the production, so these packages can be characterized as most suitable for subsequent application to packaged foods.

In general, when migrating substances from packaging to food, it is more likely to avoid migration in the case of film-forming components—e.g., migration of monomers of PET materials, etc., that are undesirable and must meet limits [71]. In contrast, the migration of active components such as polyphenols and antioxidants is desirable because they can contribute to improving the properties of packaged foods, prolong their shelf life, prevent oxidation, and thus function as a package with active properties [12].

Non-correlating results were found for antioxidant activity, i.e., in different methods (ABTS, FRAP and DPPH) the same sample did not always show the highest value of antioxidant activity, for these samples the effect of solvent is excluded, as the same solvent was used everywhere to detect migration of active substances, but the results are influenced by the conditions and the course of reactions of individual methods. For the FRAP method, the analysis is performed at low pH values (3.6) compared to the DPPH and ABTS methods, where the pH value was not adjusted. In the ABTS method, color loss is determined spectrophotometrically after the addition of an antioxidant to the blue-green chromophore ABTS^·+^, so the antioxidant reduces ABTS^·+^ to ABTS and decolorizes it [72]. The DPPH method uses a stable free radical, and in the presence of an antioxidant compound it can donate a hydrogen atom; lead to a reduction and decolorization of the dark purple solution; in the case of DPPH this radical does not always react with the same compounds as in the case of ABTS; and the stability of the ABTS solution is also much lower than that of DPPH [72,73].

## 4. Conclusions

The research showed that a film-forming solution composed of chitosan, lactic acid solution and the addition of compacted extracts prepared from plant by-products is stable in terms of zeta-potential determination. The aggregation of particles is not present, which should affect the textural properties of prepared films. Based on the results of gas barrier properties, the water vapor transmission rate increased with the addition of extracts and an opposite trend was found in measuring of oxygen permeability; based on good oxygen barrier properties the prepared films have similar values as commonly used synthetic packaging materials. Furthermore, it was found that the value of swelling degree was significantly affected by the presence of polyphenolic substances, and thus in samples with extracts of blueberry and red grape marc, the samples with the highest value of polyphenols had the lowest value of swelling degree; a good example for the protection of packaged foodstuff against water. The FTIR results analyzed by advanced processing showed the decrease in the signal of lactic acid and lactate and increase in signal specific for every plant used for extract preparation. Another interesting finding was determined by antimicrobial analysis, the films with the addition of extract from red grapes showed the formation of inhibition zones during incubation with *E. coli.* In the case of migratory techniques, the transition of bioactive compounds was confirmed, meaning that the experimentally produced edible packaging can be defined as active.

It must be emphasized that the content of polyphenols and antioxidant activity did not show high values compared to previous research, but this can be further investigated in the continuation of research, where the method of preparation of extracts can be modified to increase the amount of these active substances and also apply packaging directly to food and determine their effect on the shelf life of food.

## Figures and Tables

**Table 1:** Composition of prepared films.

Sample	Composition
CH_L_	1.5 g chitosan + 1% lactic acid + glycerol
5CH_LBO_	1.5 g chitosan + 1% lactic acid + 5% blueberry extract + glycerol
10CH_LBO_	1.5 g chitosan + 1% lactic acid + 10% blueberry extract + glycerol
20CH_LBO_	1.5 g chitosan + 1% lactic acid + 20% blueberry extract + glycerol
5CH_LPE_	1.5 g chitosan + 1% lactic acid + 5% parsley extract + glycerol
10CH_LPE_	1.5 g chitosan + 1% lactic acid + 10% parsley extract + glycerol
20CH_LPE_	1.5 g chitosan + 1% lactic acid + 20% parsley extract + glycerol
5CH_LHR_	1.5 g chitosan + 1% lactic acid + 5% red grapes extract + glycerol
10CH_LHR_	1.5 g chitosan + 1% lactic acid + 10% red grapes extract + glycerol
20CH_LHR_	1.5 g chitosan + 1% lactic acid + 20% red grapes extract + glycerol

**Table 2:** Zeta-potential of film forming solution.

Sample	Zeta Potential (mV)	Conductivity (mS/cm)
CH_L_	33.84 ± 0.72	2.008 ± 0.005 ^ac^
5CH_LBO_	34.58 ± 2.14 ^ac^	1.980 ± 0.020 ^c^
10CH_LBO_	32.26 ± 1.07 ^abe^	1.808 ± 0.008 ^b^
20CH_LBO_	31.44 ± 1.36 ^abe^	1.922 ± 0.008 ^d^
5CH_LPE_	34.52 ± 1.36 ^acd^	2.050 ± 0.001 ^ef^
10CH_LPE_	30.62 ± 2.09 ^be^	2.000 ± 0.007 ^ac^
20CH_LPE_	36.68 ± 0.32 ^c^	2.084 ± 0.006 ^g^
5CH_LHR_	31.98 ± 1.83 ^abe^	1.920 ± 0.008 ^d^
10CH_LHR_	32.22 ± 2.02 ^abe^	1.996 ± 0.011 ^ac^
20CH_LHR_	31.30 ± 2.32 ^de^	2.029 ± 0.022 ^af^

Letters in superscript indicate statistically significant (*p* < 0.05) differences between rows.

**Table 3:** Thickness of chitosan films.

Sample	Thickness (mm)
CH_L_	0.174 ± 0.013
5CH_LBO_	0.176 ± 0.046
10CH_LBO_	0.172 ± 0.040
20CH_LBO_	0.214 ± 0.022
5CH_LPE_	0.196 ± 0.030
10CH_LPE_	0.188 ± 0.019
20CH_LPE_	0.180 ± 0.017
5CH_LHR_	0.206 ± 0.055
10CH_LHR_	0.186 ± 0.019
20CH_LHR_	0.210 ± 0.029

**Table 4:** Textural properties expressed as strength (MPa) and breaking strain (%).

Sample	Strength (MPa)	Breaking Strain (%)
CH_L_	0.06 ± 0.04	122.34 ± 11.89 ^acd^
5CH_LBO_	0.04 ± 0.01 ^a^	116.01 ± 15.25 ^ad^
10CH_LBO_	0.09 ± 0.03	107.72 ± 9.89 ^a^
20CH_LBO_	0.06 ± 0.01	102.08 ± 7.26 ^a^
5CH_LPE_	0.04 ± 0.01 ^a^	136.67 ± 18.18
10CH_LPE_	0.08 ± 0.03	113.60 ± 30.36
20CH_LPE_	0.10 ± 0.02 ^b^	149.16 ± 3.54 ^ce^
5CH_LHR_	0.04 ± 0.01 ^a^	142.99 ± 13.18 ^def^
10CH_LHR_	0.05 ± 0.01 ^a^	182.41 ± 17.94 ^be^
20CH_LHR_	0.09 ± 0.04	118.10 ± 9.80 ^af^

Letters in superscript indicate statistically significant (*p* < 0.05) differences between rows.

**Table 5:** Gas barrier properties expressed as water vapor transmission rate and oxygen permeability.

Sample	Water Vapor Permeability (23 °C, 85 % RH) (g/m^2^ d)	Oxygen Transmission Rate (23 °C, 0 % RH) (mL/m^2^ d 0.1 MPa)
CH_L_	1235.8 ± 42.1 ^a^	15.1 ± 0.8
5CH_LBO_	1270.0 ± 25.3 ^a^	11.4 ± 1.1
10CH_LBO_	1327.4 ± 57.6 ^ac^	8.7 ± 0.7
20CH_LBO_	1485.1 ± 72.8 ^de^	4.1 ± 1.0
5CH_LPE_	1356.2 ± 37.6 ^acde^	12.6 ± 0.5
10CH_LPE_	1467.3 ± 50.9 ^e^	9.8 ± 1.3
20CH_LPE_	1719.4 ± 22.3 ^b^	6.5 ± 0.5
5CH_LHR_	1318.8 ± 66.3 ^ac^	13.7 ± 1.4
10CH_LHR_	1422.4 ± 47.0 ^cde^	10.3 ± 0.4
20CH_LHR_	1724.6 ± 30.8 ^b^	5.2 ± 0.9

Letters in superscript indicate statistically significant (*p* < 0.05) differences between rows.

**Table 6:** Water content, solubility and swelling degree of chitosan.

Sample	Water Content (%)	Solubility (%)	Swelling Degree (%)
CH_L_	17.11 ± 1.59 ^a^	48.02 ± 5.00	287.60 ± 77.36
5CH_LBO_	14.50 ± 2.80	46.90 ± 1.50	122.26 ± 26.72 ^acd^
10CH_LBO_	13.55 ± 0.57 ^bc^	45.12 ± 1.52 ^a^	93.23 ± 6.39 ^af^
20CH_LBO_	17.84 ± 1.95 ^a^	45.49 ± 2.21	74.28 ± 21.55 ^agf^
5CH_LPE_	13.83 ± 0.95 ^bc^	45.49 ± 2.21	151.91 ± 20.18 ^d^
10CH_LPE_	14.83 ± 0.83 ^ac^	49.59 ± 4.58 ^ac^	150.53 ± 18.23 ^cd^
20CH_LPE_	12.73 ± 0.38 ^b^	46.06 ± 0.80 ^b^	118.28 ± 21.99 ^fcd^
5CH_LHR_	15.76 ± 0.48 ^a^	49.77 ± 1.67	108.56 ± 19.01 ^fcde^
10CH_LHR_	15.45 ± 0.96 ^ac^	49.79 ± 2.96 ^bc^	69.91 ± 13.74 ^eag^
20CH_LHR_	15.56 ± 2.21	45.87 ± 1.65 ^ac^	43.99 ± 7.12 ^g^

Letters in superscript indicate statistically significant (*p* < 0.05) differences between rows

**Table 7:** Total polyphenol content of chitosan films.

Sample	TPC (mg Gallic Acid/g)
CH_L_	0.12 ± 0.01 ^a^
5CH_LBO_	0.47 ± 0.00 ^b^
10CH_LBO_	0.49 ± 0.00 ^b^
20CH_LBO_	0.85 ± 0.00 ^cf^
5CH_LPE_	0.09 ± 0.00 ^a^
10CH_LPE_	0.32 ± 0.07 ^abde^
20CH_LPE_	0.47 ± 0.02 ^bd^
5CH_LHR_	0.43 ± 0.00 ^d^
10CH_LHR_	0.72 ± 0.00 ^eg^
20CH_LHR_	0.96 ± 0.05 ^fg^

Letters in superscript indicate statistically significant (*p* < 0.05) differences between rows.

**Table 8:** Determination of rosmarinic acid, chlorogenic acid and epigallocatechin in chitosan films with addition of blueberry, parsley and red grapes extracts.

Sample	Rosmarinic Acid (µg/g)	Chlorogenic Acid (µg/g)	Epigallocatechin (mg/g)
CH_L_	6.17 ± 0.13 ^a^	0.00 ± 0.00 ^ad^	0.00 ± 0.00
5CH_LBO_	4.52 ± 0.20 ^b^	14.62 ± 0.85 ^b^	0.49 ± 0.43 ^ca^
10CH_LBO_	4.85 ± 0.28 ^b^	21.04 ± 0.41 ^e^	1.22 ± 0.01 ^dc^
20CH_LBO_	8.56 ± 0.16 ^c^	78.09 ± 6.12 ^f^	3.12 ± 0.01 ^e^
5CH_LPE_	7.76 ± 0.12 ^d^	0.00 ± 0.00 ^d^	0.00 ± 0.00
10CH_LPE_	11.15 ± 0.05 ^e^	6.62 ± 0.39 ^c^	2.68 ± 0.46 ^ged^
20CH_LPE_	20.63 ± 0.90 ^f^	5.14 ± 4.76 ^bcde^	2.33 ± 1.13
5CH_LHR_	4.16 ± 0.63 ^ab^	0.00 ± 0.00 ^d^	0.00 ± 0.00 ^ba^
10CH_LHR_	3.64 ± 0.57 ^ab^	0.00 ± 0.00 ^d^	0.00 ± 0.00 ^ba^
20CH_LHR_	6.97 ± 3.60 ^abcde^	0.00 ± 0.00 ^d^	1.31 ± 0.16 ^hcg^

Letters in superscript indicate statistically significant (*p* < 0.05) differences between rows.

**Table 9:** Determination of epicatechingallate and epicatechine in chitosan films with the addition of blueberry, parsley and red grapes extracts.

Sample	Epicatechin Gallate (mg/g)	Epicatechin (mg/g)
CH_L_	0.00 ± 0.00	0.00 ± 0.00
5CH_LBO_	0.00 ± 0.00	8.02 ± 0.19 ^a^
10CH_LBO_	3.02 ± 0.36 ^a^	12.94 ± 0.18 ^b^
20CH_LBO_	5.18 ± 0.03 ^c^	24.75 ± 0.02 ^c^
5CH_LPE_	0.00 ± 0.00	0.00 ± 0.00
10CH_LPE_	165.46 ± 1.29 ^d^	1.47 ± 0.04 ^d^
20CH_LPE_	182.78 ± 4.50 ^d^	2.74 ± 0.06 ^e^
5CH_LHR_	3.09 ± 0.11 ^a^	4.62 ± 0.07 ^f^
10CH_LHR_	4.62 ± 0.76 ^bac^	4.68 ± 0.47 ^ef^
20CH_LHR_	76.47 ± 0.69 ^e^	9.70 ± 0.22 ^g^

Letters in superscript indicate statistically significant (*p* < 0.05) differences between rows.

**Table 10:** Antioxidant properties of chitosan films.

Sample	FRAP (μmol Trolox/g)	ABTS (%)	DPPH (%)
CH_L_	0.12 ± 0.07 ^a^	0.09 ± 0.02 ^a^	1.03 ± 0.07 ^ai^
5CH_LBO_	0.50 ± 0.02 ^b^	0.48 ± 0.12 ^ae^	2.16 ± 0.05 ^ln^
10CH_LBO_	1.15 ± 0.01 ^cf^	0.71 ± 0.07 ^e^	2.75 ± 0.01 ^mo^
20CH_LBO_	2.45 ± 0.06 ^d^	0.76 ± 0.08 ^e^	5.39 ± 0.06 ^e^
5CH_LPE_	0.92 ± 0.08 ^cf^	0.95 ± 0.04 ^e^	1.17 ± 0.56 ^abcdflop^
10CH_LPE_	1.06 ± 0.37 ^bcf^	1.02 ± 0.13 ^de^	1.89 ± 0.63
20CH_LPE_	1.23 ± 0.08 ^cf^	1.47 ± 0.02 ^cd^	2.48 ± 3.43 ^fhr^
5CH_LHR_	1.02 ± 0.04 ^c^	2.29 ± 0.08 ^b^	1.18 ± 0.32 ^inopr^
10CH_LHR_	1.26 ± 0.04 ^f^	3.35 ± 0.18 ^f^	1.64 ± 0.03 ^bgp^
20CH_LHR_	1.89 ± 0.04 ^e^	4.23 ± 0.27 ^g^	2.03 ± 0.86 ^fhjlm^

Letters in superscript indicate statistically significant (*p* < 0.05) differences between rows.

**Table 11:** Migration—total polyphenols content.

Sample	TPC (mg Gallic Acid/mL)	TPC (mg Gallic Acid/g)
CH_L_	0.002 ± 0.000 ^a^	0.12 ± 0.01 ^a^
5CH_LBO_	0.005 ± 0.000 ^c^	0.47 ± 0.00 ^b^
10CH_LBO_	0.008 ± 0.000 ^d^	0.49 ± 0.00 ^b^
20CH_LBO_	0.010 ± 0.000 ^e^	0.85 ± 0.00 ^cf^
5CH_LPE_	0.004 ± 0.000 ^b^	0.09 ± 0.00 ^a^
10CH_LPE_	0.010 ± 0.000 ^f^	0.32 ± 0.07 ^abde^
20CH_LPE_	0.011 ± 0.000 ^g^	0.47 ± 0.02 ^bd^
5CH_LHR_	0.011 ± 0.000 ^e^	0.43 ± 0.00 ^d^
10CH_LHR_	0.012 ± 0.000 ^g^	0.72 ± 0.00 ^eg^
20CH_LHR_	0.016 ± 0.000 ^h^	0.96 ± 0.05 ^fg^

Letters in superscript indicate statistically significant (*p* < 0.05) differences between rows

**Table 12:** Migration–antioxidant activity.

Sample	FRAP (μmol Trolox/mL)	ABTS (%)	DPPH (%)
CH_L_	0.0112 ± 0.0004 ^a^	2.60 ± 0.40 ^a^	34.22 ± 1.25 ^a^
5CH_LBO_	0.0111 ± 0.0002 ^a^	2.70 ± 0.04 ^c^	51.62 ± 3.00 ^cadg^
10CH_LBO_	0.0137 ± 0.0005 ^c^	3.10 ± 0.08 ^d^	54.95 ± 0.50 ^d^
20CH_LBO_	0.0210 ± 0.0002 ^d^	3.81 ± 0.19 ^e^	70.07 ± 0.97 ^b^
5CH_LPE_	0.0163 ± 0.0002 ^e^	3.73 ± 0.04 ^e^	34.45 ± 2.56 ^ead^
10CH_LPE_	0.0152 ± 0.0002 ^b^	4.86 ± 0.05 ^b^	53.33 ± 0.34 ^fcd^
20CH_LPE_	0.0170 ± 0.0001 ^e^	4.94 ± 0.05 ^b^	58.75 ± 0.06 ^g^
5CH_LHR_	0.0153 ± 0.0005 ^b^	2.67 ± 0.08 ^c^	41.48 ± 3.00 ^acd^
10CH_LHR_	0.0129 ± 0.0005 ^cb^	3.24 ± 0.08 ^d^	52.00 ± 7.60
20CH_LHR_	0.0248 ± 0.0004 ^f^	4.35 ± 0.05 ^f^	53.15 ± 3.64 ^gcdb^

Letters in superscript indicate statistically significant (*p* < 0.05) differences between rows.

## Data Availability

Not applicable.

## References

[1] Salgado P.R., Ortiz C.M., Musso Y.S., Di Giorgio L., Mauri A.N. (2015). Edible films and coatings containing bioactives. Curr. Opin. Food Sci..

[2] Wang Q., Liu W., Tian B., Li D., Liu C., Jiang B., Feng Z. (2020). Preparation and characterization of coating based on protein nanofibers and polyphenol and application for salted duck egg yolks. Foods.

[3] Balti R., Mansour M.B., Zayoud N., Le Balc’h R., Brodu N., Arhaliass A., Massé A. (2020). Active exopolysaccharides based edible coatings enriched with red seaweed (*Gracilaria gracilis*) extract to improve shrimp preservation during refrigerated storage. Food Bioscience.

[4] Hu Y., Shi L., Ren Z., Hao G., Chen J., Weng W. (2021). Characterization of emulsion films prepared from soy protein isolate at different preheating temperatures. J. Food Eng..

[5] Kaczmarek B., Owczarek A., Nadolna K., Sionkowska A. (2019). The film-forming properties of chitosan with tannic acid addition. Mater. Lett..

[6] Kumar M.N.R. (2000). A review of chitin and chitosan applications. React. Funct. Polym..

[7] Hirano S., Gebelein C.G., Carraher C.E. (1995). Industrial Biotechnological Polymers.

[8] Le Y., Anand S.C., Horrocks A.R. (1997). Recent developments in fibres and materials for wound management. Indian J. Fibre Text..

[9] Nair K.R., Madhavan P. (1984). Chitosan for removal of mercury from water. Fish. Technol..

[10] Ma Q., Ren Y., Gu Z., Wang L. (2017). Developing an intelligent film containing Vitis amurensis husk extracts: The effects of pH value of the film-forming solution. J. Clean. Prod..

[11] Vermeiren L., Devlieghere F., van Beest M., de Kruijf N., Debevere J. (1999). Developments in the active packaging of foods. Trends Food Sci. Technol..

[12] Espitia P.J.P., Du W.X., de Jesús Avena-Bustillos R., Soares N.D.F.F., McHugh T.H. (2014). Edible films from pectin: Physical-mechanical and antimicrobial properties—A review. Food Hydrocoll..

[13] Lalnunthari C., Devi L.M., Badwaik L.S. (2019). Extraction of protein and pectin from pumpkin industry by-products and their utilization for developing edible film. J. Food Sci. Technol..

[14] Tkaczewska J., Jamróz E., Kulawik P., Morawska M., Szczurowska K. (2019). Evaluation of the potential use of a carp (*Cyprinus carpio*) skin gelatine hydrolysate as an antioxidant component. Food Funct..

[15] Jancikova S., Jamróz E., Kulawik P., Tkaczewska J., Dordevic D. (2019). Furcellaran/gelatin hydrolysate/rosemary extract composite films as active and intelligent packaging materials. Int. J. Biol. Macromol..

[16] Torres-León C., Vicente A.A., Flores-López M.L., Rojas R., Serna-Cock L., Alvarez-Pérez O.B., Aguilar C.N. (2018). Edible films and coatings based on mango (var. Ataulfo) by-products to improve gas transfer rate of peach. Lwt.

[17] Faria A., Oliveira J., Neves P., Gameiro P., Santos-Buelga C., de Freitas V., Mateus N. (2005). Antioxidant properties of prepared blueberry (*Vaccinium myrtillus*) extracts. J. Agric. Food Chem..

[18] Nakatani N. (1997). Antioxidants from spices and herbs. Natural Antioxidants: Chemistry, Health Effects, and Applications.

[19] Rodríguez-Díaz R.C., Aguilar-Caballos M.P., Gómez-Hens A. (2006). Determination of some hydroxybenzoic acids and catechins in white wine samples by liquid chromatography with luminescence detection. J. Sep. Sci..

[20] Downey M.O., Dokoozlian N.K., Krstic M.P. (2006). Cultural practice and environmental impacts on the flavonoid composition of grapes and wine: A review of recent research. Am. J. Enol. Viticult..

[21] Charles D.J. (2012). Parsley. Handbook of Herbs and Spices.

[22] Hänsel R., Keller K., Rimpler H., Schneider G. (1994). Hagers Handbuch Der Pharmazeutischen Praxis. 5. 6.

[23] Tauferova A., Pospiech M., Javurkova Z., Tremlova B., Dordevic D., Jancikova S., Tesikova K., Zdarsky M., Vitez T., Vitezova M. (2021). Plant Byproducts as Part of Edible Coatings: A Case Study with Parsley, Grape and Blueberry Pomace. Polymers.

[24] Souza V.G.L., Fernando A.L., Pires J.R.A., Rodrigues P.F., Lopes A.A., Fernandes F.M.B. (2017). Physical properties of chitosan films incorporated with natural antioxidants. Ind. Crop. Prod..

[25] Mlynáriková K., Samek O., Bernatová S., Růžička F., Ježek J., Hároniková A., Šiler M., Zemánek P., Holá V. (2015). Influence of culture media on microbial fingerprints using Raman spectroscopy. Sensors.

[26] Tomadoni B., Cassani L., Ponce A., Moreira M.D.R., Agüero M.V. (2016). Optimization of ultrasound, vanillin and pomegranate extract treatment for shelf-stable unpasteurized strawberry juice. LWT-Food Sci. Technol..

[27] Gómez-Estaca J., Bravo L., Gómez-Guillén M.C., Alemán A., Montero P. (2009). Antioxidant properties of tuna-skin and bovine-hide gelatin films induced by the addition of oregano and rosemary extracts. Food Chem..

[28] Behbahani B.A., Shahidi F., Yazdi F.T., Mortazavi S.A., Mohebbi M. (2017). Use of Plantago major seed mucilage as a novel edible coating incorporated with Anethum graveolens essential oil on shelf life extension of beef in refrigerated storage. Int. J. Biol. Macromol..

[29] Thaipong K., Boonprakob U., Crosby K., Cisneros-Zevallos L., Byrne D.H. (2006). Comparison of ABTS, DPPH, FRAP, and ORAC assays for estimating antioxidant activity from guava fruit extracts. J. Food Compos. Anal..

[30] Adilah A.N., Jamilah B., Noranizan M.A., Hanani Z.N. (2018). Utilization of mango peel extracts on the biodegradable films for active packaging. Food Packag. Shelf Life.

[31] European Commission (2011). Commission Regulation (EU) No 10/2011 of 14 January 2011 on plastic materials and articles intended to come into contact with food. Off. J. Eur. Union.

[32] Cheung R.C.F., Ng T.B., Wong J.H., Chan W.Y. (2015). Chitosan: An update on potential biomedical and pharmaceutical applications. Mar. Drugs.

[33] Larsson M., Hill A., Duffy J. (2012). Suspension stability; why particle size, zeta potential and rheology are important. Ann. Trans. Nord. Rheol. Soc..

[34] Kanmani P., Rhim J.W. (2014). Antimicrobial and physical-mechanical properties of agar-based films incorporated with grapefruit seed extract. Carbohyd. Polym..

[35] Kanatt S.R., Rao M.S., Chawla S.P., Sharma A. (2012). Active chitosan–polyvinyl alcohol films with natural extracts. Food Hydrocoll..

[36] Jridi M., Boughriba S., Abdelhedi O., Nciri H., Nasri R., Kchaou H., Kaya M., Sebai H., Zouari N., Nasri M. (2019). Investigation of physicochemical and antioxidant properties of gelatin edible film mixed with blood orange (*Citrus sinensis*) peel extract. Food Packag. Shelf Life.

[37] Park S.K., Rhee C.O., Bae D.H., Hettiarachchy N.S. (2001). Mechanical properties and water-vapor permeability of soy-protein films affected by calcium salts and glucono-δ-lactone. J. Agric. Food Chem..

[38] Siripatrawan U., Vitchayakitti W. (2016). Improving functional properties of chitosan films as active food packaging by incorporating with propolis. Food Hydrocoll..

[39] Kalaycıoğlu Z., Torlak E., Akın-Evingür G., Özen İ., Erim F.B. (2017). Antimicrobial and physical properties of chitosan films incorporated with turmeric extract. Int. J. Biol. Macromol..

[40] Jancikova S., Dordevic D., Jamroz E., Behalova H., Tremlova B. (2020). Chemical and Physical Characteristics of Edible Films, Based on κ-and ι-Carrageenans with the Addition of Lapacho Tea Extract. Foods.

[41] Kittur F., Kumar K., Tharanathan R. (1998). Functional packaging properties of chitosan films. Z. Lebensm. Unters. Forsch..

[42] Kołodziejska I., Piotrowska B. (2007). The water vapour permeability, mechanical properties and solubility of fish gelatin–chitosan films modified with transglutaminase or 1-ethyl-3-(3-dimethylaminopropyl) carbodiimide (EDC) and plasticized with glycerol. Food Chem..

[43] Cazón P., Vázquez M., Velazquez G. (2018). Novel composite films based on cellulose reinforced with chitosan and polyvinyl alcohol: Effect on mechanical properties and water vapour permeability. Polym. Test..

[44] Bedane A.H., Eić M., Farmahini-Farahani M., Xiao H. (2015). Water vapor transport properties of regenerated cellulose and nanofibrillated cellulose films. J. Membr. Sci..

[45] Robertson G.L. (2012). Food Packaging: Principles and Practice.

[46] Rambabu K., Bharath G., Banat F., Show P.L., Cocoletzi H.H. (2019). Mango leaf extract incorporated chitosan antioxidant film for active food packaging. Int. J. Biol. Macromol..

[47] Srinivasa P.C., Ramesh M.N., Tharanathan R.N. (2007). Effect of plasticizers and fatty acids on mechanical and permeability characteristics of chitosan films. Food Hydrocoll..

[48] Reyes-Chaparro P., Gutierrez-Mendez N., Salas-Muñoz E., Ayala-Soto J.G., Chavez-Flores D., Hernández-Ochoa L. (2015). Effect of the addition of essential oils and functional extracts of clove on physicochemical properties of chitosan-based films. Int. J. Polym. Sci..

[49] Elsabee M.Z., Abdou E.S. (2013). Chitosan based edible films and coatings: A review. Mater. Sci. Eng. C.

[50] Rhim J.-W. (2004). Increase in water vapor barrier property of biopolymer-based edible films and coatings by compositing with lipid materials. Food Sci. Biotechnol..

[51] Wang L., Wang Q., Tong J., Zhou J. (2017). Physicochemical properties of chitosan films incorporated with honeysuckle flower extract for active food packaging. J. Food Process Eng..

[52] Ojagh S.M., Rezaei M., Razavi S.H., Hosseini S.M.H. (2010). Development and evaluation of a novel biodegradable film made from chitosan and cinnamon essential oil with low affinity toward water. Food Chem..

[53] Bourbon A.I., Pinheiro A.C., Cerqueira M.A., Rocha C.M., Avides M.C., Quintas M.A., Vicente A.A. (2011). Physico-chemical characterization of chitosan-based edible films incorporating bioactive compounds of different molecular weight. J. Food Eng..

[54] Favaro L.I., Balcão V.M., Rocha L.K., Silva E.C., Oliveira J.M., Vila M.M., Tubino M. (2018). Physicochemical characterization of a crude anthocyanin extract from the fruits of Jussara (*Euterpe edulis Martius*): Potential for food and pharmaceutical applications. J. Brazil. Chem. Soc..

[55] Morar M.I., Fetea F., Rotar A.M., Nagy M., Semeniuc C.A. (2017). Characterization of essential oils extracted from different aromatic plants by FTIR spectroscopy. Bull. Univ. Agric. Sci. Vet. Med. Cluj Napoca. Food Sci. Technol..

[56] Ertani A., Pizzeghello D., Francioso O., Sambo P., Sanchez-Cortes S., Nardi S. (2014). Capsicum chinensis L. growth and nutraceutical properties are enhanced by biostimulants in a long-term period: Chemical and metabolomic approaches. Front. Plant Sci..

[57] Bors W., Michel C., Stettmaier K. (2001). Structure-activity relationships governing antioxidant capacities of plant polyphenols. Methods in Enzymology.

[58] Siripatrawan U., Harte B.R. (2010). Physical properties and antioxidant activity of an active film from chitosan incorporated with green tea extract. Food Hydrocoll..

[59] Moradi M., Tajik H., Rohani S.M.R., Oromiehie A.R., Malekinejad H., Aliakbarlu J., Hadian M. (2012). Characterization of antioxidant chitosan film incorporated with Zataria multiflora Boiss essential oil and grape seed extract. LWT-Food Sci. Technol..

[60] Ruiz-Navajas Y., Viuda-Martos M., Sendra E., Perez-Alvarez J.A., Fernández-López J. (2013). In vitro antibacterial and antioxidant properties of chitosan edible films incorporated with Thymus moroderi or Thymus piperella essential oils. Food Control.

[61] Saberi B., Vuong Q.V., Chockchaisawasdee S., Golding J.B., Scarlett C.J., Stathopoulos C.E. (2017). Physical, barrier, and antioxidant properties of pea starch-guar gum biocomposite edible films by incorporation of natural plant extracts. Food Bioprocess Technol..

[62] Lapornik B., Prošek M., Wondra A.G. (2005). Comparison of extracts prepared from plant by-products using different solvents and extraction time. J. Food Eng..

[63] Crozier A., Yokota T., Jaganath I.B., Marks S., Saltmarsh M., Clifford M.N. (2006). Secondary metabolites in fruits, vegetables, beverages and other plant based dietary components. Plant Secondary Metabolites: Occurrence, Structure and Role in the Human Diet.

[64] Kim K.W., Thomas R.L. (2007). Antioxidative activity of chitosans with varying molecular weights. Food Chem..

[65] Xie W., Xu P., Liu Q. (2001). Antioxidant activity of water-soluble chitosan derivatives. Bioorg. Med. Chem. Lett..

[66] Negro C., Tommasi L., Miceli A. (2003). Phenolic compounds and antioxidant activity from red grape marc extracts. Bioresour. Technol..

[67] Wong P.Y., Kitts D.D. (2006). Studies on the dual antioxidant and antibacterial properties of parsley (*Petroselinum crispum*) and cilantro (*Coriandrum sativum*) extracts. Food Chem..

[68] Katalinić V., Možina S.S., Skroza D., Generalić I., Abramovič H., Miloš M., Ljubenkov I., Piskernik S., Pezo I., Terpinc P. (2010). Polyphenolic profile, antioxidant properties and antimicrobial activity of grape skin extracts of 14 Vitis vinifera varieties grown in Dalmatia (Croatia). Food Chem..

[69] Baydar N.G., Özkan G., Sağdiç O. (2004). Total phenolic contents and antibacterial activities of grape (*Vitis vinifera* L.) extracts. Food Control.

[70] Farag R.S., Daw Z.Y., Abo-Raya S.H. (1989). Influence of some spice essential oils on *Aspergillus parasiticus* growth and production of aflatoxins in a synthetic medium. J. Food Sci..

[71] Arvanitoyannis I.S., Bosnea L. (2004). Migration of substances from food packaging materials to foods. Crc Rev. Food Sci..

[72] Alam M.N., Bristi N.J., Rafiquzzaman M. (2013). Review on in vivo and in vitro methods evaluation of antioxidant activity. Saudi Pharm. J..

[73] Stratil P., Klejdus B., Kubáň V. (2007). Determination of phenolic compounds and their antioxidant activity in fruits and cereals. Talanta.

